# Pre-Hospital ECG E-Transmission for Patients with Suspected Myocardial Infarction in the Highlands of Scotland

**DOI:** 10.3390/ijerph110202346

**Published:** 2014-02-21

**Authors:** Gordon F. Rushworth, Charlie Bloe, H. Lesley Diack, Rachel Reilly, Calum Murray, Derek Stewart, Stephen J. Leslie

**Affiliations:** 1 Highland Clinical Research Facility, Centre for Health Science, Old Perth Road, Inverness IV2 3JH, UK; E-Mail: gordon.rushworth@nhs.net; 2 Cardiac Unit, Raigmore Hospital, Old Perth Road, Inverness IV2 3UJ, UK; E-Mail: charles.bloe@nhs.net; 3 School of Pharmacy and Life Sciences, Robert Gordon University, Riverside East, Garthdee Road, Aberdeen AB10 7GJ, UK; E-Mails: h.l.diack@rgu.ac.uk (H.L.D.); r.reilly1@rgu.ac.uk (R.R.); calummurray14@hotmail.co.uk (C.M.); d.stewart@rgu.ac.uk (D.S.); 4 Highland Campus, Centre for Health Science, University of Stirling, Old Perth Road, Inverness IV2 3UJ, UK

**Keywords:** telehealthcare, telehealth, telecardiology, ECG, pre-hospital thrombolysis, cardiac triage

## Abstract

Patients with ST elevation myocardial infarction (STEMI) require prompt treatment, best done by primary percutaneous coronary intervention (PPCI). However, for patients unable to receive PPCI, immediate pre-hospital thrombolysis (PHT) is the best alternative. Evidence indicates that diagnostic and management support for staff increases the use of PHT. This study aimed to describe the patient demographics and management of patients, to determine any potential inter-area differences in referral rates to the ECG e-transmission service and to explore the views and experiences of key staff involved in ECG e-transmission within NHS Highland. Data from 2,025 patient episodes of ECG e-transmission identified a statistically significant geographical variation in ECG e-transmission and PHT delivery. Scottish Ambulance Service (SAS) staff were more likely than GPs to deliver PHT overall, however, GPs were more likely to deliver in remote areas. Interviews with six Cardiac Care Unit (CCU) nurses and six SAS staff highlighted their positive views of ECG e-transmission, citing perceived benefits to patients and interprofessional relationships. Poor access to network signal was noted to be a barrier to engaging in the system. This study has demonstrated that a specialist triage service based on e-transmission of ECGs in patients with suspected STEMI can be implemented in a diverse geographical setting. Work is needed to ensure equity of the service for all patients.

## 1. Introduction

While mortality from coronary heart disease (CHD) is declining [1], patients with ST elevation myocardial infarction (STEMI) continue to have poor outcomes, especially if treatment is delayed. Within the Highlands region of Scotland, CHD is the primary cause of mortality, with one of the highest mortality rates in the UK for men under 75 years of age [2]; approximately 120 patients per annum have a STEMI within NHS Highland. Primary percutaneous coronary intervention (PPCI) is the evidence based treatment choice for STEMI [3] but in those situations where this cannot be delivered (e.g., due to a lack of access to a catheterisation laboratory) immediate thrombolysis is advised, preferably given in the prehospital setting (PHT) [4]. It is well recognised that short delays in treatment with PPCI [5,6,7] and thromobolysis [8,9,10,11,12,13,14] can result in increased mortality and morbidity. However, prompt treatment, requires prompt diagnosis which has been shown to be facilitated by pre-hospital ECG e-transmission [15].

### 1.1. ECG E-transmission Service

NHS Highland is geographically the largest health board in the UK and covers an area of approximately one third of Scotland’s landmass, 32,500 km^2^. However, it contains less than 4% of the population at 320,990, making the region one of the most sparsely populated areas in Europe. NHS Highland is serviced by four acute hospitals, with only one coronary care unit (CCU) and cardiac catheterisation laboratory. 

The ECG e-transmission service in Highland was set up in 2008 to improve the triage of patients presenting with symptoms of acute STEMI *i.e.*, chest pain, shortness of breath, arm/jaw/neck pain. ECGs are electronically transmitted by Scottish Ambulance Service (SAS) paramedics or General Practitioners (GP) attending patients with STEMI symptoms. If a diagnosis of STEMI is confirmed from the ECG and there are no contra-indications, CCU specialist staff will advise SAS/GP by phone to deliver PHT immediately. As at the time of this study there was no PPCI service provision within the Highlands. While this is at variance with practice for much of the rest of the UK and Europe where PPCI is first-line treatment [3], this is similar to many remote and rural areas where rurality/geographical isolation may limit access to cath lab services [16,17]. Within such areas PHT should be the principal reperfusion strategy [18]. 

The service is e-mail based using the secure national ‘nhs.net’ e-mail system which is available to all health care staff work within the National Health Service (NHS). The defibrillators used by the SAS are Phillips HeartStart MRx Monitor Defibrillator Model M3536A. This acquires the 12 lead ECG data and communicates it to a cellphone using Bluetooth^®^ which then sends this to the HeartStart 12 Lead Transfer Station (LTS) which receives, decrypts, and saves the 12-lead ECG to a database. The HeartStart 12 LTS then sends a PDF of the ECG to a designated email address which is manned 24/7/365 by CCU staff. While these devices have a computer generated diagnostic mode, the standard protocol is that the diagnosis and treatment decision by the onsite team (SAS/GP) to administer PHT is supported by CCU staff. In part this is due to a self-reported low confidence in GP ECG diagnostic skills which has previously been identified as a barrier to the use of PHT and those staff treating patients in the pre-hospital setting expressed a desire for additional training and support in ECG analysis [19]. In a further study of GP attitudes to PHT conducted in a neighbouring but less remote and rural geographical region of Scotland, only two thirds respondents reported willingness to record and interpret an ECG [20]. In order to overcome these barriers the ECG e-transmission service was established.

### 1.2. Research Aims

The aims of this study were to describe the patient demographics and management of patients, to determine any potential inter-area differences in referral rates to the ECG e-transmission service and to explore the views and experiences of key staff involved in ECG e-transmission within NHS Highland.

## 2. Methods

This was a two staged study comprising analysis of routinely collected data from ECG e-transmission records followed by qualitative interviews with CCU nurses and SAS crew members. 

### 2.1. ECG E-transmission Database

A Microsoft Access^®^ database recorded all ECG e-transmissions from January 2010 to August 2012. Anonymised data collected were: patient gender; age; presenting complaint; ECG appearance; date and time of e-ECG transmission; and transmitting ambulance station. Travel time to hospital was estimated by SAS crew members. In addition, for those patients with STEMI or LBBB, CCU staff members completed a standardised proforma on receipt of an ECG e-transmission, with details of PHT delivery (by whom and where). Data were entered into the Access database, within one week of transmission. Access database entries were exported to SPSS^®^ version 21, with reliability of sample data entry confirmed prior to analysis. 

Descriptive statistics were carried out to characterise the sample. Data are presented as mean (SD) unless otherwise stated. Chi-square was used as a test of association between patient gender/age and e-transmission; and the student-*t* test to explore differences in transmission rates/hours of transmission over time. *p* < 0.05 was considered statistically significant.

### 2.2. Qualitative Interview

Semi-structured, face-to-face interviews were conducted with a convenience sample of locally available SAS crew members within the Inverness Ambulance Station and nurses based within the Coronary Care Unit at Raigmore Hospital (tertiary treatment centre), Inverness, all of whom had experience of use of the ECG e-transmission service. The sampling frame for the qualitative interviews encompassed all SAS crew members working within Inverness Ambulance Station (n = 27) and all CCU staff nurses (n = 24) within Raigmore Hospital in Inverness. Six crew members and six nurses were invited to participate in a face-to-face interview. The interview schedule was developed based on the findings of previous research [19], with particular focus on their experiences of the service, operational aspects, advantages and disadvantages compared to previous service delivery and patient care, and future service delivery. The interview schedule was reviewed by members of the research team prior to piloting with one crew member and nurse. No changes were made post-pilot and hence the pilot data were included in the study analysis. Interviews, lasting between 10 and 20 min, were audio-recorded and transcribed verbatim. Thematic analysis was undertaken independently by two researchers. 

### 2.3. Research Governance

The study was approved by the ethical review panel of the School of Pharmacy and Life Sciences at Robert Gordon University and by the NHS Highland Research and Development Office.

## 3. Results

### 3.1. Quantitative Data Analysis

#### 3.1.1. Summary of the Study Participants

Data were collected on 2,025 ECG e-transmissions between January 2010 and August 2012. Patient demographics are given in Table 1 and compared to population data based on the Highland Council Region collected during the Scottish census 2011 [21]. More males than females were referred and referrals were more common for those patients over 70 years.

**Table 1:** Sample demographics (n = 2,025) compared to population data.

Characteristic		% (n)	Population Data	*p*-value (χ^2^)
Gender	Male	61.4 (1,243)	48.9 (113,471)	*p* < 0.00001
	Female	36.9 (748)	51.1 (118,661)	
	Missing	1.7 (34)		
Age (years)	<50	16.2 (328)	62.2 (137,061)	
	50–70	37.4 (758)	29.0 (63,963)	
	>70	43.5 (880)	8.7 (19,177)	*p* < 0.00001
	Missing	2.9 (59)		

#### 3.1.2. Variation in E-transmission Rates

**Figure 1:**
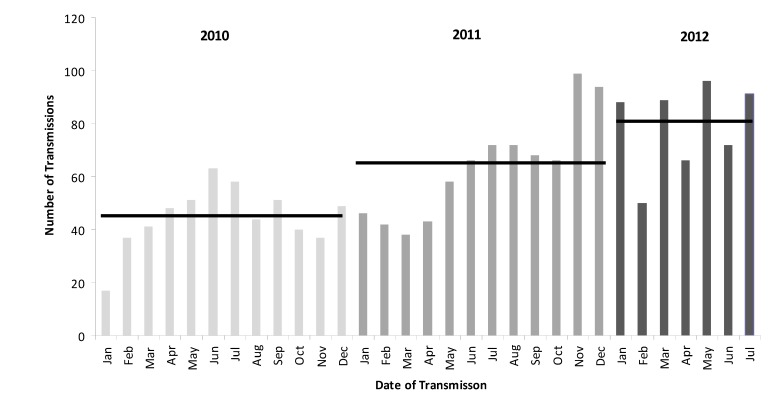
Uptake of service between January 2010 and July 2013; horizontal bars represents the mean number of transmissions per year or part thereof.

**Figure 2:**
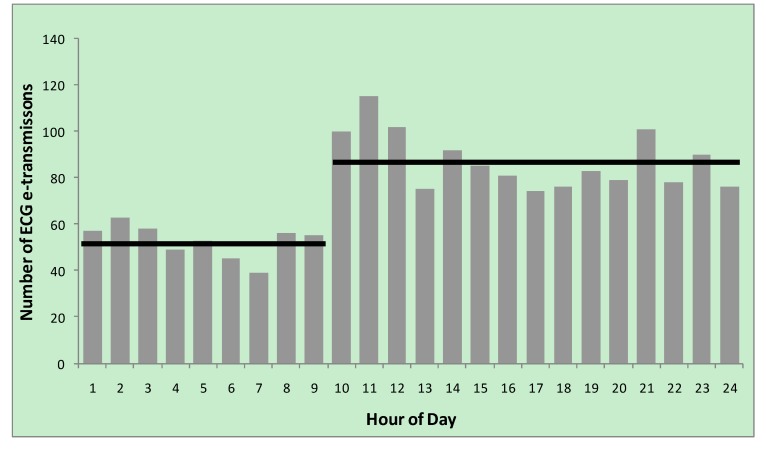
Diurnal variation in e-transmission of ECG; horizontal bars represent the mean number of transmissions for the hours spanned by the bar.

Figure 1 illustrates the increase in e-transmissions since initiation of the service in 2010 and Figure 2 e-transmissions per hour, with lowest activity between midnight and 10:00. There were highly statistically significant increases in the number of transmissions between 2010 and 2011 (n = 536, (mean n/month = 44.67; SD 11.8) vs n = 764 (mean n/month = 63.67; SD 19.6) respectively; *p* = 0.004 (one-tailed student’s *t*-test)). There was also a statistically significant increase in transmissions pro-rata between 2011 and 2012 (n = 764 ( mean n/month = 63.67; SD 19.6) *vs.* n = 552 (mean n/month = 78.86) respectively; *p* = 0.047 (one-tailed student’s *t*-test)).

There was a clear diurnal variation with fewer transmissions per hour between 12 midnight and 10 am compared with other hours (00:00–09:59 n = 475, (mean = 52.8; SD 7.3) *vs.* 10:00–23:59 n = 1,307, (mean = 87.1; SD 12.4); *p* < 0.001).

#### 3.1.3. Treatment Delivery Resulting from Transmission

Table 2 provides data on the location of the event in relation to hospital, reported ECG findings and any treatment administered. Only 40.9% of patients (123/301) with STEMI or new LBBB received thrombolysis of which only 92 (74.8%) were given PHT. The providers of PHT were usually ambulance paramedics (72, 78.3%) or GPs (14, 15.2%). Recording the reason for not giving thrombolysis was inconsistent, however in those 60 patients where a reason was reported, 32 (53.3%) were late presenters and contra-indications were noted in a further 24 (40%). 

**Table 2:** ECG e-transmission and subsequent treatment.

Parameter	Groups	% (n)
Travel time to hospital (n = 1,535)	<30 min	62.1 (954)
	31–60 min	18.1 (277)
	61–90 min	10.4 (160)
	>90 min	9.4 (144)
If STEMI/presumed new LBBB, was thrombolysis given? (n = 301)	Yes	40.9 (123)
	No	59.1 (178)
ST Elevation Detail (n = 147)	Inferior STEMI	55.8 (82)
	Anterior STEMI	40.1 (59)
	Lateral STEMI	4.1 (6)
Location thrombolysis given (n = 123)	Pre-hospital	74.8 (92)
	CCU	8.9 (11)
	A&E	4.9 (6)
	Other	1.6 (2)
	Missing	9.8 (12)
PHT given by (n = 92)	Paramedic	78.3 (72)
	GP	15.2 (14)
	Missing	6.5 (6)
If given PHT admitted to (n = 92)	CCU	80.4 (74)
	A&E	44 (8)
	Caithness General	16.7 (3)
	Broadford Hospital	11.1 (2)
	Stornoway Hospital	11.1 (2)
	Medical Receiving	5.6 (1)
	Aberdeen Royal CCU	5.6 (1)
	Belford Hospital	5.6 (1)

Of the 92 patients receiving PHT, 17 (18.5%) patients were taken to an acute hospital without a CCU or PPCI facility. 

Table 3 shows that almost one fifth (17.6%) of patients living within 30 mins of hospital were not given PHT, but instead treatment was delayed until the patient was transferred to hospital care. Where thrombolysis was given, it was more likely given in the pre-hospital opposed to hospital setting 73 *vs.* 11 respectively. PHT delivery was mainly delivered by paramedics for those patients within 90 min travel time and by GPs for those living >90 min from hospital. 

**Table 3:** Proximity to hospital at time of event *vs.* location and administrator of thrombolysis (n = 84).

Travel Time to Hospital (mins)	Thrombolysis Administration % (n) *			Total % (n)
	Pre-hospital Thrombolysis		Hospital Thrombolysis	
	Paramedic	GP	Hospital Staff	
<30	76.5 (39)	5.9 (3)	17.6 (9)	26.2 (51)
31–60	88.2 (15)	11.8 (2)	0 (0)	20.2 (17)
61–90	100 (7)	0 (0)	0 (0)	8.3 (7)
>90	11.1 (1)	66.7 (6)	22.2 (2)	10.7 (9)
Sub-total	73.8 (62)	13.1 (11)	13.1 (11)	100 (84)

Note: * Details on who administered thrombolysis in relation to proximity to hospital only available for 84 out of 123 due to missing data.

**Figure 3:**
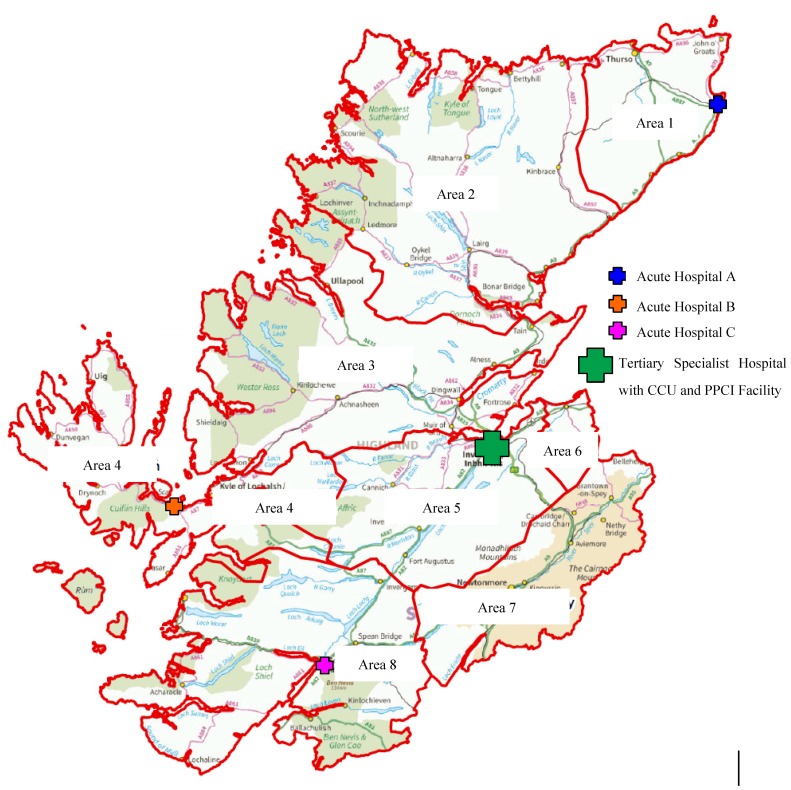
Map of Highland areas and acute hospitals.

Note: Source—Highland Council based on National Records of Scotland mid-year estimates 2011.

There were significant differences in the referral rates from different geographical areas of the Highlands in respect of ECG e-transmissions (Table 4), with remote areas 1, 4 and 8 having lowest use per head of population. Figure 3 illustrates that while there are acute hospitals in these three areas which receive medical admissions, none have access to PPCI facilities (see Figure 3). 

### 3.2. Qualitative Data

#### 3.2.1. Summary of the Study Participants

Twelve members of staff (six CCU nurses and six SAS) agreed to be interviewed. Demographics are given in Table 5, highlighting the diversity of experience in terms of clinical experience generally and specifically relating to e-ECG transmission.

**Table 4:** E-transmissions and PHT by area (population figures as National Records of Scotland mid-year estimates 2011: [22]).

Highland Area	Population of area (n = 222,370)	E-transmissions *			Supported PHT delivered		
		N (n = 1,395)	Per 10,000 population	Range	N (n = 66)	Per 10,000 population	Range
1	25,160	30	11.9		3	1.19	
2	13,520	129	95.4		4	2.96	
3	51,450	473	91.9	11.9–95.4	21	4.08	0.52–7.29
4	12,680	32	25.2		3	2.37	
5	74,950	515	68.7		19	2.54	
6	12,340	70	56.7		9	7.29	
7	12,890	114	88.4		6	4.65	
8	19,290	32	16.6		1	0.52	
			Mean 62.7			Mean 2.97	

Note: * Details on e-transmissions in relation to Highland area only available for 1,395 out of 2,025 due to missing data.

**Table 5:** Summary of the demographic data of the study participants and their involvement with the telemetry service.

Participant Code	Gender	Years of Experience	Frequency of Direct Involvement in Reception/ Transmission of an ECG to CCU via Telemetry Service	Frequency of Administration of PHT (SAS only)
CCU 1	Male	28	Daily	
CCU 2	Female	23	Daily	
CCU 3	Female	10	Daily	
CCU 4	Female	5	Daily	
CCU 5	Female	12	Daily	
CCU 6	Female	14	Daily	
SAS 1	Male	18	On average six times per week	Three times in the last year
SAS 2	Male	9	Varies-sent four last week	Once in two years
SAS 3	Female	<1	On average weekly	Assisted once
SAS 4	Male	20	Monthly	Four in the last year
SAS 5	Male	9	Once or twice a week	Once in the last year
SAS 6	Male	13	Once a week	Three in the last year

All were very positive of their experiences, citing benefits to patient care and practitioners. Patients were thought to benefit through earlier intervention:

*“…the benefit to the patient is a much earlier intervention with a consequence of less damage to the heart and therefore better outlook for them.”*

*[CCU1]*

Additionally, patients were triaged more rapidly to specialist care, who in turn had prior warning of patient admission:

*“If someone is acutely unwell and they present as a STEMI then they can come directly to ourselves whereas before normally they might have just been transferred straight to A&E and it’s better if we see the ECG because we can tell if the person is having an acute MI they come directly here and it saves a lot of time as well.”*

*[CCU3]*

Furthermore, advantages arose from nurses’ clinical awareness of individual patients:

*“…it’s great because they can give us more insight and they can look up patient records. What we’re seeing, we might see something, eh, not normal but it’s normal for that patient because they have already seen the previous ECGs.”*

*[SAS2]*

Practitioner benefits were improved communication, notably interprofessional communication:

*“…it’s good contact, a discussion with another professional so we can actually, you know, feed off each other as well during the process of passing over the information.”*

*[SAS4]*

*“I think we all work together as a team. Paramedics value our support and we are very open to receiving the calls…”*

*[CCU3]*

*“Since it was first introduced I think there is probably a better understanding between the two of us now…there is more of a connection now I think between the staff in the CCU and ourselves. Eh, I definitely think there is a better working ethic in there.”*

*[SAS6]*

The need for role definition and effective interprofessional team working was highlighted by one nurse:

*“when you are doing telemetric advice, you’re using someone at the end of a phone as your eyes and ears and they’re looking at the patient trying to convey their clinical opinion to you and whereas all you’re seeing is the ECG.”*

*[CCU1]*

While few cited major disadvantages or service barriers were described, almost all raised the issue of potentially poor telephone signals, particularly in remote and rural areas:

*“I think one of the barriers is technical, we have a history of problems with just technical issues with the signals not coming through.”*

*[CCU2]*

While one nurse noted that the service could detract from the care of other patients:

*“I suppose it takes up more nursing time and takes us away from our patients…”*

*[CCU3]*

Another described some differences between the clinical guidelines employed by crew members compared to nurses:

*“A national issue but the JR-CALC guidelines are always an issue because their thrombolysis guidelines differ from us.”*

*[CCU5]*

## 4. Discussion

### 4.1. Summary of Main Findings

This study demonstrated that remote ECG triage by a tertiary specialist centre can be completed successfully using digital technology as a means of providing support and advice to healthcare professionals attending patients with suspected STEMI. The quantitative data provided evidence that the use of the service has expanded over time. However, despite a diagnosis of STEMI/LBBB, a relatively low number of patients (<50%) were given thrombolysis and this figure was even lower for PHT (<40%). Where thrombolysis was given, it was more likely to be given pre-hospital, although there appeared to be a relationship between PHT and distance of patients from hospital. Those more distant were less likely to receive PHT and furthermore, lower ECG e-transmission rates per population suggesting that a large proportion of patients with STEMI were not treated by using the ECG e-transmission service. The provider of PHT was more likely to be SAS if <90min, or a GP if >90min from hospital. 

It is possible that those paramedics servicing larger concentrations of population are more likely to have greatest experience of administering PHT. Prior work within the Highlands has provided evidence that any experience in delivering PHT is associated with a greater likelihood of administering PHT in the future [19].

Members of staff, both in the tertiary specialist centre or distal care providers, were positive about their experiences of using ECG e-transmission triage. All groups thought there were benefits to patients with some additional benefits of the triage system being improved interprofessional working and communication and a pre-admission knowledge of a patient for specialist staff. No major barriers to use of the service were noted by either group, however, poor phone signal was noted to be a problem in some areas.

### 4.2. Strengths and Limitations

#### 4.2.1. Quantitative Data

We researched one geographical region within Scotland and hence findings may lack external validity in relation to the rest of the UK and further afield. While most of the data were routinely collected, we required staff to document additional data on treatments given, which may have introduced an element of bias and there were several instances of missing data. Furthermore, patients who were admitted directly to hospitals via other routes (e.g., by family) were not included in this study which could in part explain the variation in the use of the ECG e-transmission service in more remote areas.

#### 4.2.2. Qualitative Data

Convenience sampling was employed to identify and interview CCU and SAS staff and data saturation may not have been achieved with the low numbers interviewed. 

### 4.3. Interpretation of Findings

#### 4.3.1. Remote Area Service Variation

One concern arising from these results is the geographical variation seen between areas of the Highlands, both in terms of ECG e-transmission rates and PHT delivery. This raises the possibility of inequity of access to specialist triage for STEMI and provision of evidence based treatment. While areas 1, 4 and 8 had the lowest numbers of transmissions, these data should be interpreted with caution. Although the uptake of the specialist ECG e-transmission service and hence recorded PHT delivery would appear to be lower in these areas it may be that a scoop and run practice prevails in these areas and patients are taken directly to the local hospital. This may still be an inappropriate care pathway as these local acute hospitals have no access to PPCI facilities or CCU and transporting patients to these locations would result in a delay to the delivery of thrombolysis.

#### 4.3.2. Seasonal Variation

Despite the gradual increase in the use of this service, there was a marked seasonal variation with more e-transmissions in the summer and over the Christmas and New Year period. This may be due, in part to the influx of tourists to the Highlands of Scotland, an issue to which service planners should be alerted. 

#### 4.3.3. Diurnal Variation

Data highlight a diurnal variation in service use. While chest pain and MI rates are highest during the immediate waking period, the dramatic increase in transmissions seen in our study (almost treble between 7:00 and 11:00) suggests that there may be other unidentified factors. One potential factor may be those patients experiencing chest pain overnight being reticent to call for help outside office hours, which may indicate a need for increased awareness of the general public around the presentation and management of those experiencing symptoms suggestive of myocardial infarction.

#### 4.3.4. Facilitators and Barriers to Uptake

CCU and SAS staff highlighted the patient benefits and improved interprofessional communication since the introduction of the ECG e-transmission service. While these responses are encouraging, there is a need for effective channels of communication and definition of role which should be reinforced as the service is extended, particularly in the more remote areas. While we did not research the GPs’ perspectives of the service, we have previously shown that in urban areas, some GPs (particularly those who were geographically close to an Emergency Department) expressed the opinion that emergency care was not their responsibility [19]. However, findings from our study indicate that GPs almost exclusively delivered PHT when >90mins from a specialist tertiary centre, highlighting the role of the remote and rural GP as being distinctly different from their urban counterparts. 

Reliable technology, including access to a signal for the e-transmission of the ECG is an absolute requirement for the delivery of the service. Transmission failures were identified as a barrier to the service and hence optimal management for suspected MI in some patients. There is a need to explore and enhance the technology available for SAS staff in terms of the ability to access any network signal available to transmit data.

One further barrier to delivering PHT was the differences in guidelines for CCU and SAS staff in terms of patient inclusion criteria for pre-hospital opposed to hospital thrombolysis. This requires further investigation, with data from a previous study stating that no contraindications to PHT were present in 21% of patients admitted to hospital with acute MI who were not given PHT [23]. 

### 4.4. Future Directions and Studies

Further research is required to identify any explanation as to why particular geographical areas appear to be have a lower use of the e-transmission service and to understand and hence minimise the impact of transmission failure due to poor mobile phone signal. Mapping the patient journey is warranted from the point of presentation of symptoms, their attempts to access to NHS services, GPS locations of where ECG e-transmissions were sent PHT, any subsequent hospital treatment and outcomes (clinical, economic and humanistic). There is a need to research and further develop the service from the viewpoint of all stakeholders, including patients, GPs and carers, NHS 24, management, and those members of SAS staff with less or no experience of the service. 

## 5. Conclusion

This study has demonstrated that a specialist triage service based on e-transmission of ECGs in patients with suspected STEMI can be implemented in a diverse geographical setting. Work is needed to ensure equity of the service for all patients as there would appear to be differences in the frequency of referrals per head of capita from some geographical areas in Highland. 
